# Optimization of Nano-encapsulation on Neonatal Porcine Islet-like Cell Clusters Using Polymersomes

**DOI:** 10.1186/s11671-021-03512-3

**Published:** 2021-03-31

**Authors:** Sang Hoon Lee, Hyun-Ouk Kim, Jung-Taek Kang

**Affiliations:** 1 MGENPLUS Biotechnology Research Institute, Mgenplus Co., Ltd, Seoul, 06688 Republic of Korea; 2 Department of Biotechnology and Bioengineering, Kangwon National University, Gangwon-do, Chuncheon, 24341 Republic of Korea; 3 Biohealth-Machinery Convergence Engineering, Kangwon National University, Gangwon-do, Chuncheon, 24341 Republic of Korea

**Keywords:** Porcine islets, Polymersomes, Nano-encapsulation

## Abstract

Researches proving methods for nano-encapsulation of neonatal porcine islet-like cell clusters (NPCCs) using polymersomes (PSomes) formed using polymers of polyethylene glycol-block-poly lactide. Herein, our studies present efficient nano-encapsulation procedure with minimal damage and loss of NPCCs.We used N-hydroxysuccinimide (NHS) on the N-terminal of PSomes to induce binding of amine groups in the extracellular matrix surrounding NPCCs. F-10 culture medium with bovine serum albumin was used in the nano-encapsulation procedure to minimize damage and loss of NPCCs. Finally, we induced cross-linking between bifunctional PSomes (NHS-/NH_2_-PSomes). F-10 culture medium containing 0.25% BSA with pH of 7.3 minimized the damage and loss of NPCCs after nano-encapsulation as compared with using basic HBSS buffer (pH 8.0). Also, we induced the efficient nano-encapsulation through conjugation of PSomes using bifunctional PSomes (NHS-/NH_2_-PSomes).

**Supplementary Information:**

The online version contains supplementary material available at 10.1186/s11671-021-03512-3.

## Introduction

The use of allo-islet transplantation in the treatment of type 1 diabetes is limited owing to lack of suitable donors. Instead, there is a gradual increase in the use of animal islets in xeno-islet transplantation, with pigs emerging as optimal donor species [1]. When pigs are used as donors during transplantation, separate islets can be used, based on the age of the pigs. Often, neonatal porcine islet-like cell clusters (NPCCs) are preferred over adult porcine islets (APIs) owing to their affordability and ease of isolation. In addition, NPCCs can proliferate gradually after transplantation, prolonging their function in vivo [1, 2]. However, when NPCCs are transplanted into human or non-human primate (NHP) portal veins, interspecies variations can cause immune reactions such as instant blood-mediated inflammatory reaction (IBMIR) or hyperacute rejection, leading to early graft loss [3]. To solve this problem, encapsulation of NPCCs that can inhibit various immune responses is required. There are three types of encapsulation: macro-, micro-, and nano-encapsulation. Macro-encapsulation uses a device containing islets, which is then implanted around blood vessels to release insulin through a semipermeable membrane in response to blood glucose levels. Micro-encapsulation packs a small number of islets into a porous capsule. Although these encapsulations can protect islets from immune rejection, side effects such as membrane collapse or thrombus generation have been reported in in vivo trials. The islets also disturb the flow of hormones, nutrients, or oxygen due to increase in diffusion distance. Nano-encapsulation is the strategy of cell surface modification inducing attachment between cells and exogenous proteins, mainly polyethylene glycol (PEG), in blood transfusion [4].

Nano-encapsulation using PEG has been widely used as a modification method for improving efficacy and physicochemical properties of target proteins or peptides [5]. In particular, nano-encapsulation of islets may have inhibitory effects in response to immune cell attack and antibody recognition. PEG is widely used for cell coating because of its biocompatible properties such as non-immunogenicity, antigen masking, and non-fouling effect [6]. Among the nanoparticles used for nano-encapsulation of NPCCs, "polymersomes" (PSomes) based on PEG-block-poly lactide (PEG-b-PLA) are the most suitable because they are stable and simple to modify; they can also incorporate both hydrophilic and hydrophobic reagents in their assembly [7, 8]. The surface modification of islets using polymers (containing PEG) is accomplished through covalent or non-covalent binding between the extracellular matrix (ECM) of the islet and the functional group conjugated polymer [9].

In previous studies, a basic Hank’s balanced salt solution (HBSS, pH 8.0) was used as the islet nano-encapsulation reaction buffer because of its ability to facilitate N- hydroxysuccinimide (NHS)-NH_2_ binding [10–12]. However, to minimize cellular damage to NPCCs during nano-encapsulation, we used F-10 medium (NPCCs culture medium) with physiological pH (pH 7.3). In addition, because preserving the quantity of NPCCs after nano-encapsulation is important for transplantation of the correct amount of cells, we added bovine serum albumin (BSA), which coats the bottom of cell culture dish with a long chain polymer, to increase recovery rate [13]. In our previous study, the nano-encapsulation of NPCCs with PSomes was carried out via single functional groups, such as NHS or NH_2_, that are used to induce covalent binding or electrostatic interaction with ECM of NPCCs, respectively. However, as binding affinity decreases over time, strategies are needed to increase binding efficiency [14]. PSome having bifunctional groups can be used for candidate to increase coating efficiency due to their ability to aggregate. Therefore, we induced a cross-linking between PSomes containing bifunctional groups (NHS-/NH_2_-PSome) that can bind not only to the ECM of NPCCs, but also to each PSome through covalent interaction or electrostatic interaction, thus increasing nano-encapsulation efficiency.

In this study, we investigated the possible application of the nano-encapsulation on NPCCs through optimized method in the field of porcine islets xeno-transplantation.

### Materials and Methods

#### Animals

All animal experiments were approved by the Institutional Animal Care and Use Committee of the Institute of MGENPLUS co. ltd. (#2019–1), and all procedures were carried out in accordance with guidelines set out by the committee. Surgery was performed under general anesthesia, and efforts were taken to ensure the animals experienced minimal pain. Pigs were killed prior to pancreatectomy.

#### Isolation of Neonatal Porcine Islet-like Cell Clusters (NPCCs)

NPCCs were isolated from 3- to 5-day-old piglets. Briefly, piglets were anesthetized using ketamine (10 mg/kg, Yuhan, Seoul, Korea) and xylazine hydrochloride (1 mg/kg, Rompun; Bayer Korea, Seoul, Korea) injection into the femoral muscle and then killed by injecting potassium chloride (Sigma-Aldrich, MO, USA) into the heart. The pancreas was exposed through an abdominal incision, harvested, and immersed in Hank’s balanced salt solution (HBSS, Biosesang, Gyeonggi-do, Korea) with 8.3 mM sodium bicarbonate, 10 mM N-(2-Hydroxyethyl)piperazine-N′-(2-ethanesulfonic acid) (HEPES) (Sigma-Aldrich, MO, USA), and 0.5% antibiotic–antimycotic (Biowest, MO, USA). The pancreas was chopped into 1–2 mm^3^ fragments and digested in collagenase type V (1 mg/ml, Sigma- Aldrich, MO, USA) in HBSS for 10 min. Cold HBSS containing 10% fetal bovine serum (FBS) (Biowest, MO, USA) was added to digested pancreas tissue to stop enzyme activity. Digested pancreas tissues were washed in HBSS, and after resuspension, the tissues were filtered through a pluriStrainer 500 μm (pluriSelect, Leipzig, Germany) and washed in HBSS. Finally, NPCCs were seeded and cultured in 5% CO_2_ at 37 °C in F-10 medium (Gibco, CA, USA) supplemented with 0.25% bovine serum albumin (BSA) (genDepot, TX, USA), 10 mM nicotinamide, 10 mM D-glucose, 2 mM L-glutamine, 2 mM calcium chloride dihydrate, 50 μM isobutylmethylxanthine (IBMX), 20 μg/ml ciprofloxacin (Sigma-Aldrich, MO, USA), and 1% antibiotic–antimycotic. NPCCs were cultured for 5 days [15], with 10 nM exendin-4 (Prospec, Ness-Ziona, Israel) being added to the culture media every day.

#### In Vitro Assessment of NPCCs and Nano-encapsulated NPCCs

After culture, the number of NPCCs was counted as islet equivalent (IEQ) using eyepiece reticle in ocular. Viability was assessed using acridine orange (AO, 0.67 μM, Sigma-Aldrich, MO, USA) and propidium iodide (PI, 75 μM, Sigma-Aldrich, MO, USA) staining. To perform the glucose-stimulated insulin secretion (GSIS) assay, 20–30 NPCCs were picked and pre-incubated with a low D-glucose (2.8 mM) concentration in Krebs–Ringer bicarbonate buffer (KRBB) for 1 h. NPCCs were then incubated with low D-glucose (2.8 mM) in KRBB buffer for 1 h followed by high D-glucose solution (28.0 mM) in KRBB for 1 h. Supernatants were collected to measure insulin secretion under low and high glucose concentrations [11]. The amount of insulin secreted from each sample was measured using a Human/Canine/Porcine Insulin Quantikine ELISA Kit (R&D systems, MN, USA). Stimulation index (SI) was calculated by dividing the insulin quantities at high glucose (28.0 mM) by that at low glucose (2.8 mM) concentrations.

#### Preparation of Polymersome (PSome)

To prepare PSomes, either N-hydroxysuccinimide-poly (ethylene glycol)-block-poly (lactide) copolymers (10 mg/ml, NHS-PEG-b-PLA) or amine-poly (ethylene glycol)-block-poly (lactide) copolymers (10 mg/ml, NH_2_-PEG-b-PLA; Nanosoft polymers, NC, USA) were dissolved in 1 ml of dimethyl sulfoxide (DMSO, Sigma-Aldrich, MO, USA). In addition to prepare bifunctional PSome, each copolymer (NHS or NH_2_-PEG-b-PLA) dissolved in DMSO was mixed in proportions. Distilled water (DH2O) was added to the polymer solution to make a final concentration of 1 mg/ml. The polymer solution was sonicated in an ultrasonicator (Sea han ultrasonic, Seoul, Korea) for 5 min. 1,1′-Dioctadecyl-3,3,3′,3′-tetramethylindodicarbocyanine, 4-chlorobenzenesulfonate salt (DiD; Biotium, CA, USA) was added to the polymer solution during sonication to enable visualization. Finally, the mixture was dialyzed in DH2O for 3 days.

#### Nano-encapsulation

The PSome was diluted in nano-encapsulation reaction buffer (either HBSS (pH 7.3 or pH 8.0) or plain F-10 media without supplements (pH 7.3 or pH 8.0), with or without 0.25% BSA). Nano-encapsulation was performed by adding the PSome to NPCCs in culture media. To do this, 10,000 IEQs of NPCCs were seeded in a 6-well cell culture dish (SPL, Gyeonggi-do, Korea), and diluted PSome added to the NPCCs and incubated in 5% CO_2_ at 37 °C for 1 h. A negative control (NC) group (non-coated NPCCs without PSomes) was incubated under the same condition as the experimental group. After incubation, the nano-encapsulated NPCCs were harvested and cultured in F-10 culture media.

#### Efficiency of Nano-encapsulated NPCCs

The DiD-conjugated PSome nano-encapsulated NPCCs were visualized using either fluorescence microscopy (Leica, Wetzlar, Germany) or confocal laser scanning microscopy (CLSM; Carl Zeiss, Oberkochen, Germany). The intensity of DiD-conjugated PSome-bound NPCCs was quantified by computing the mean fluorescence intensity (MFI) using ImageJ software (NIH, Bethesda, USA). Nuclei in cell were counterstained with 4′,6-diamidino-2-phenylindole (DAPI).

#### Polymersome Permeability Assay

The NHS-PSome nano-encapsulated NPCCs in F-10 or F-10 (0.25% BSA) were incubated with fluorescein isothiocyanate (FITC)-conjugated dextran of varying molecular weights (10, 20, 70, and 250 kDa) for 2 h. The penetration of FITC-conjugated dextran into NHS-PSome nano-encapsulated NPCCs was confirmed for each molecular weight via a confocal laser scanning microscope.

#### Polymersome Cell Viability Assay

The NHS-PSome nano-encapsulated THP-1 (human monocytic cell line) in RPMI 1640 (Biowest, MO, USA) was incubated for 1 h. The viability of NHS-PSome nano-encapsulated THP-1 was measured according to the protocol presented in MTT cell proliferation assay kit (iNtRon Biotechnology, Seongnam- si, Korea).

#### Statistical Analysis

Unpaired t-test was performed in GraphPad Prism 6.0. The statistical significance was expressed as *, ** ***, and **** indicating the *P* value of ≤ 0.05, ≤ 0.01, ≤ 0.001 and ≤ 0.0001.

## Results

### Culture and Functional Assessment of NPCCs

NPCCs were cultured for 5 days, and quality controls, including viability and GSIS, were performed. The total number of NPCCs was 21,014.0 IEQ/g/pancreas. The viability, using AO/PI staining, was 89.9%. GSIS, which was performed to confirm the responsiveness of NPCCs to glucose concentration, gave an average stimulation index (SI) of 2.3 (Table 1, Additional file 1: Fig. S1).

**Table 1:** The yields, viability and functionality of NPCCs at 5 days after isolation

No	NPCCs yield (IEQ/gram/pancreas weight)\	NPCCs yield (IEQ/pancreas)	Viability (%)	SI
1	23,326.0	41,986.8	91.8	2.3
2	21,219.8	37,134.7	88.2	3.6
3	18,805.0	32,909.0	89.0	2.2
4	22,855.7	39,997.5	93.0	1.2
5	18,863.4	46,215.3	87.5	2.0
Mean	21,014.0 ± 1912.3	39,648.7 ± 4482.2	89.9 ± 2.1	2.3 ± 0.7

### PSome Concentration Required for Efficient Nano-encapsulation of NPCCs

**Fig. 1:**
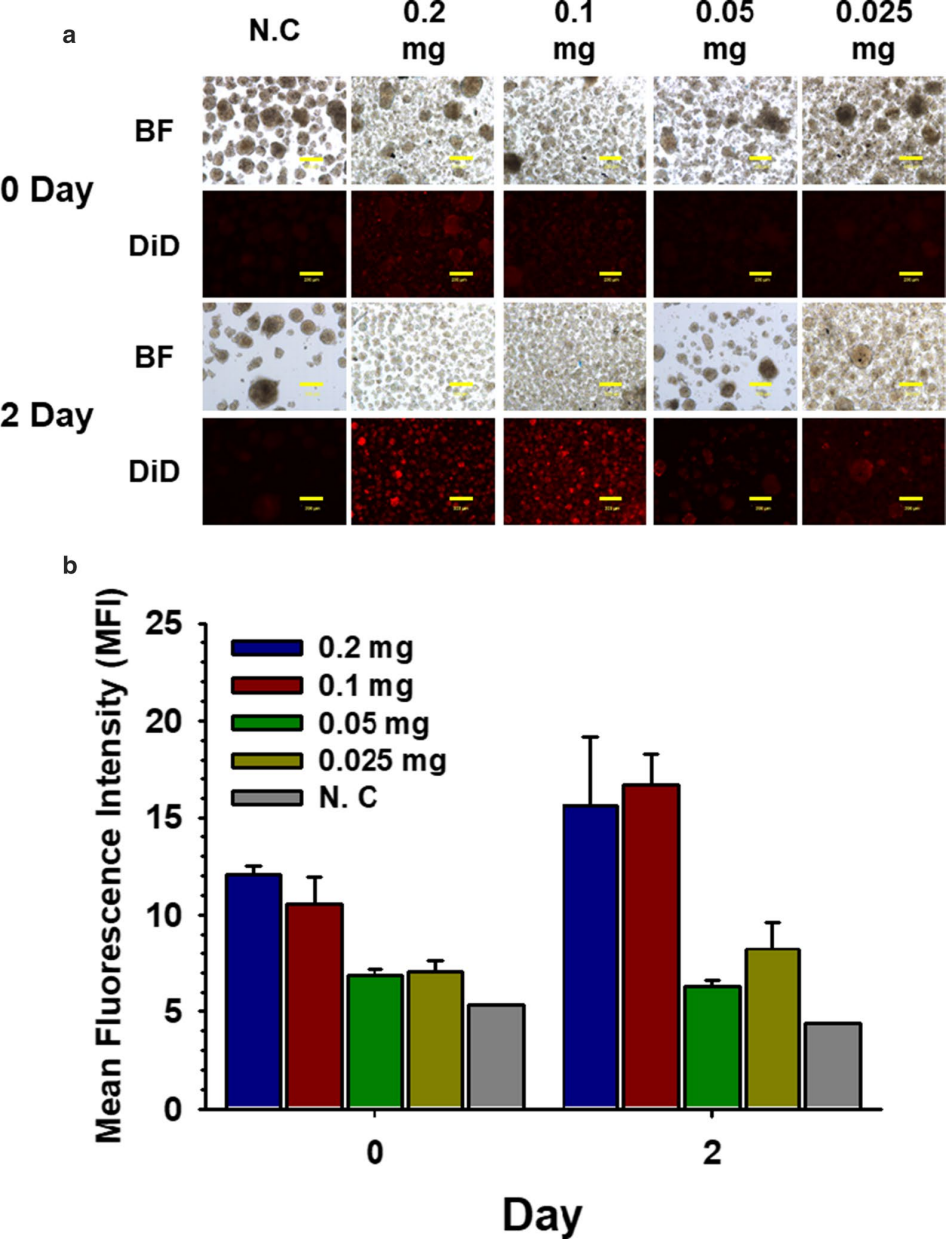
Optimization of PSome concentration for effective nano-encapsulation at 0 and 2 days. Optimization of PSome concentration for effective nano-encapsulation. **a** DiD-loaded NHS-PSome was treated in NPCCs at various concentration (BF; bright field). The scale bars represent 200 μm. **b** MFI of DiD-loaded PSome nano-encapsulated NPCCs (*n* = 3) and NC control (*n* = 1)

To determine the concentration of PSome required for efficient nano-encapsulation, we added varying concentrations of NHS-PSome to the NPCCs. NHS-PSome was stocked at a concentration of 1 mg/ml in DH_2_O and diluted at 1:5, 1:10, 1:20, and 1:40 to give final concentrations ranging from 0.2 to 0.025 mg/ml. Nano-encapsulation efficiency was measured by MFI of DiD-loaded PSome nano-encapsulated NPCCs. The 0.1 mg/ml (1:10 dilution) final concentration showed the highest fluorescence intensity at 2 days post-nano-encapsulation (Fig. 1a and b) and subsequent nano-encapsulation of NPCCs was conducted at this concentration.

### Improved Efficiency of Nano-encapsulation in F-10 Media with Physiological pH

**Fig. 2:**
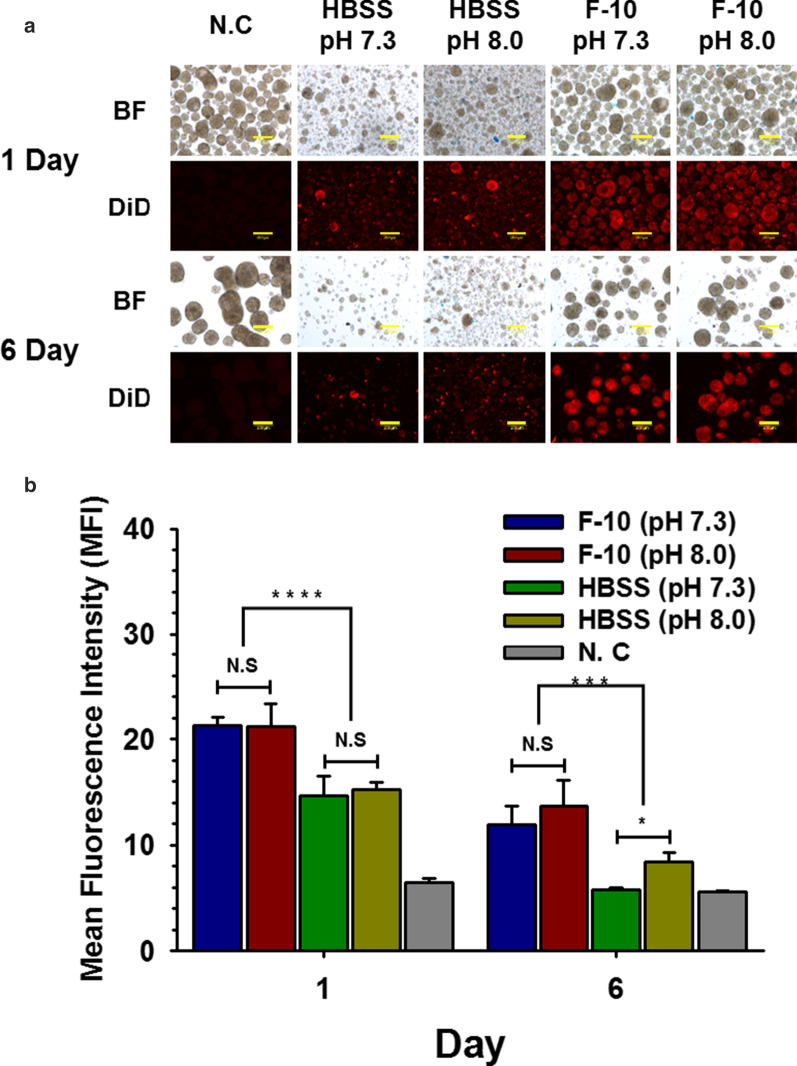
Comparisons of coating efficiency of nano-encapsulation in various reaction buffers at 1 and 6 days. Comparisons of coating efficiency of nano-encapsulation in various reaction buffers. **a** Nano-encapsulated NPCCs in HBSS (pH 7.3 and pH 8.0) or F-10 (pH 7.3 and pH 8.0) using DiD-conjugated NHS-PSome (BF; bright field). The scale bars represent 200 um; **b** MFI of nano-encapsulated NPCCs in HBSS (pH 7.3 and pH 8.0) or F-10 (pH 7.3 and pH 8.0) using DiD-conjugated NHS-PSome (all groups; *n* = 3). Data represent the mean ± S.D. *p < 0.05, ***p < 0.001 and ****p < 0.0001 versus other groups

Nano-encapsulation of pancreatic islets (containing NPCCs) is often performed in basic HBSS buffer (pH 8.0 or above) to enhance the binding affinity between NH2 in ECM of islets and NHS conjugated in polymer. However, nano-encapsulation in basic HBSS buffer can potentially damage NPCCs. Thus, to minimize the damage of NPCCs and determine the effect of pH on NHS- NH_2_ binding, NPCCs were nano-encapsulated through NHS-PSome in HBSS buffers or plain F-10 culture media (used in this study to culture NPCCs) with pH 7.3 (physiological) or pH 8.0 (basic), respectively. When NPCCs were nano-encapsulated in F-10, the normal morphology of NPCCs was maintained (Fig. 2a), and the efficiency of nano-encapsulation based on MFI was significantly increased compared with that in the HBSS group regardless of pH at days 0 and 6 (Fig. 2b). Although HBSS groups showed significant difference between pH 7.3 and 8.0 on day 6, the intensity of nano-encapsulation was significantly decreased compared with that in the F-10 group. Thus, we used physiological F-10 culture medium (pH 7.3) as the nano-encapsulation reaction buffer in subsequent experiments to minimize the potential damage of NPCCs.

### Increased Recovery Rate of NPCCs After Nano-encapsulation

**Fig. 3:**
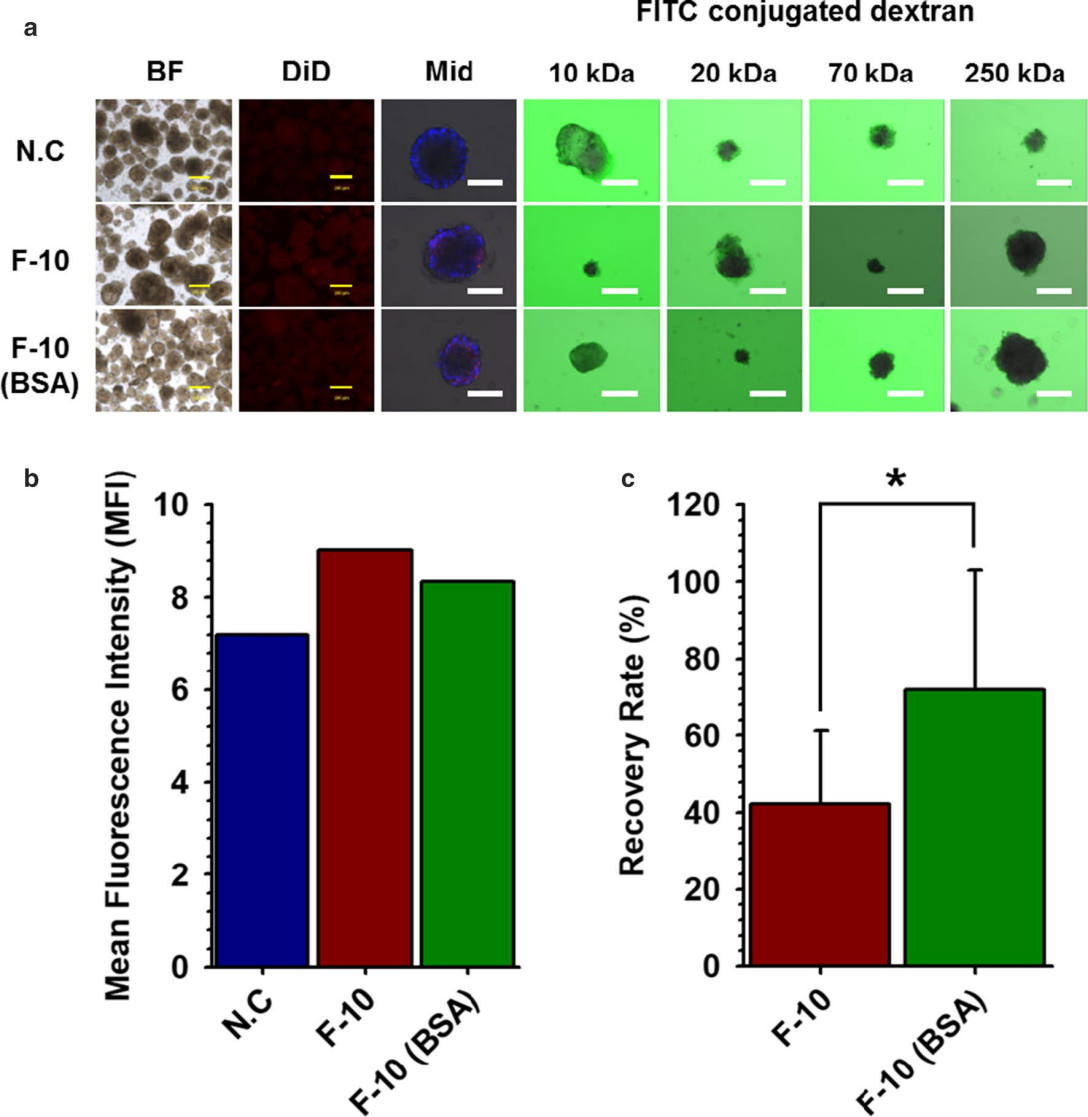
Comparisons of coating efficiency after 0.25% BSA addition in nano-encapsulation reaction buffers for increase the recovery rate. Comparisons of coating efficiency after 0.25% BSA addition in nano-encapsulation reaction buffers for increase the recovery rate. **a** Coating efficiency and selective permeability of nano-encapsulated NPCCs with NHS-PSome in F-10 or F-10 with 0.25% BSA (BF; bright field, Mid; middle image using CLSM of DiD-conjugated NHS-PSome nano-encapsulated NPCCs, FITC-conjugated dextran; middle image using CLSM of FITC-conjugated dextran in NHS-PSome nano-encapsulated NPCCs). Blue in Mid represent the cell through DAPI staining. Scale bars are 200 (BF and DiD) and 100 (Mid and FITC-conjugated dextran) μm; **b** MFI of nano-encapsulated NPCCs using DiD-conjugated NHS-PSome; **c** Recovery rate of NPCCs after nano-encapsulation in F-10 (*n* = 12) or F-10 with 0.25% BSA (*n* = 12). Data represent the mean ± S.D. *p < 0.05 versus F-10

**Fig. 4:**
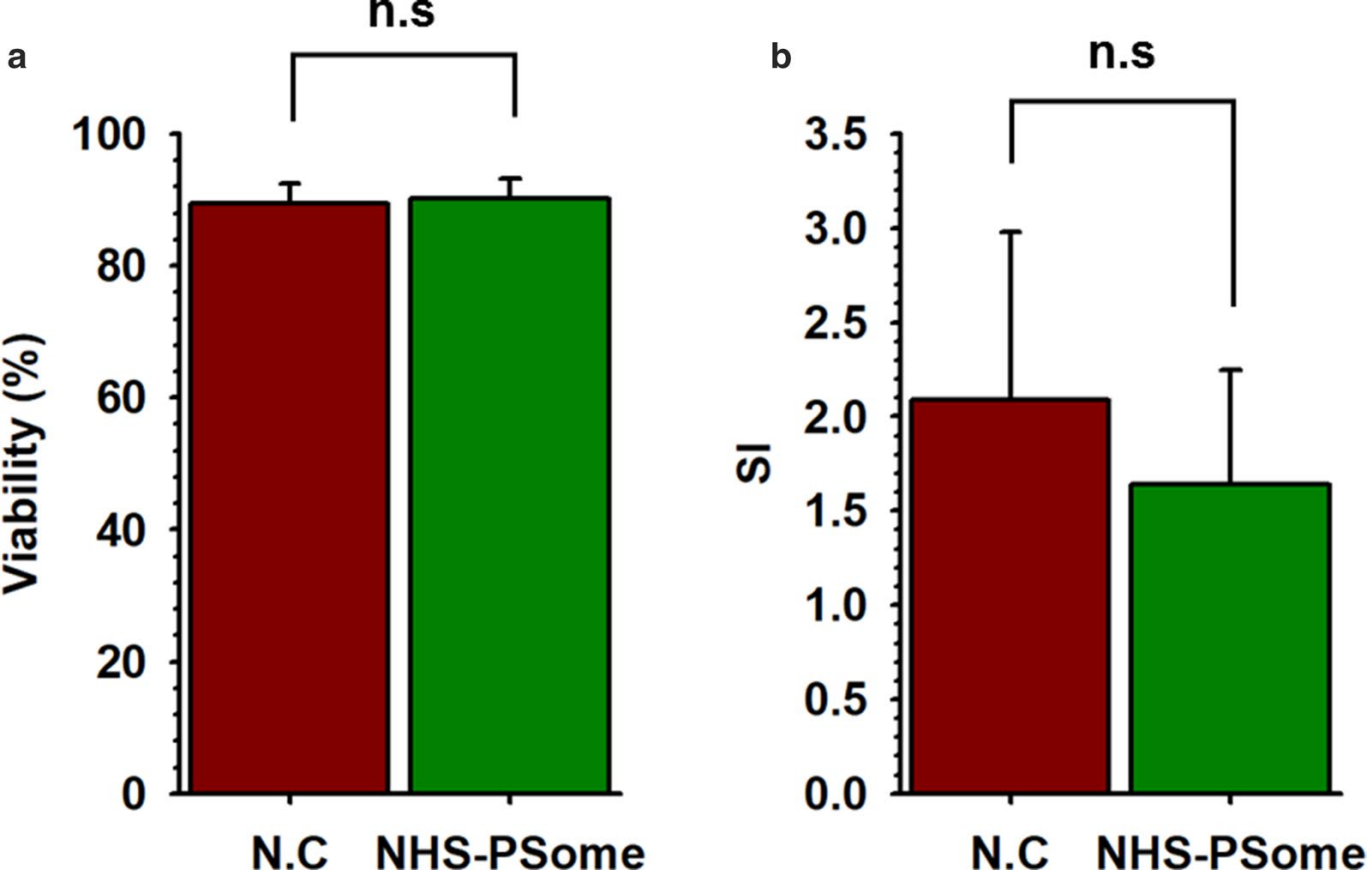
Viability and functionality of NHS-PSome nano-encapsulated NPCCs using F-10 with 0.25% BSA. Viability and functionality of NHS-PSome nano-encapsulated NPCCs using F-10 with 0.25% BSA. **a** Viability of NHS-PSome nano-encapsulated NPCCs (*n* = 6) and NC control (*n* = 6); **b** SI of NHS-PSome nano-encapsulated NPCCs (*n* = 5) and NC control (*n* = 5). Data represent the mean ± S.D

Although the cellular damage of NPCCs was minimized in F-10, the amount of NPCCs collected after nano-encapsulation was markedly reduced. To solve this problem, we added 0.25% BSA to F-10 medium during culture and nano-encapsulation of NPCCs using NHS-PSome. We initially confirmed whether adding 0.25% BSA into F-10 medium affects the coating efficiency and the selective permeability, allowing the passage of small molecules (10 and 20 kDa FITC-conjugated dextran) while blocking larger molecules (70 and 250 kDa FITC-conjugated dextran), as an essential function of PSome. As a result, the conformal coating was shown in images of CLSM (Fig. 3a, Mid) and selective permeability was maintained normally (Fig. 3b, FITC-conjugated dextran) in NHS-PSome nano-encapsulated NPCCs with F-10 containing 0.25% BSA compared to F-10, although MFI was slightly reduced (Fig. 3b). The amounts of NPCCs collected after nano-encapsulation showed significantly higher recovery rate (71.9%) in F-10 with 0.25% BSA than in F-10 without BSA (42.3%) (Fig. 3c). Viability (NC: 89.5%, NHS-PSome: 90.3%) and glucose-stimulated insulin secretion (NC: 2.1, NHS-PSome: 1.6) of NPCCs in F-10 with 0.25% BSA were also maintained after nano-encapsulation (Fig. 4a, b, Additional file 1: Fig. S2). Our results indicate that the addition of 0.25% BSA can significantly enhance the recovery rate of NPCCs after nano-encapsulation and did not affect the coating efficiency or function of PSome.

## Enhanced Stability of Nano-encapsulation Through Cross-linking PSomes

**Scheme 1.**
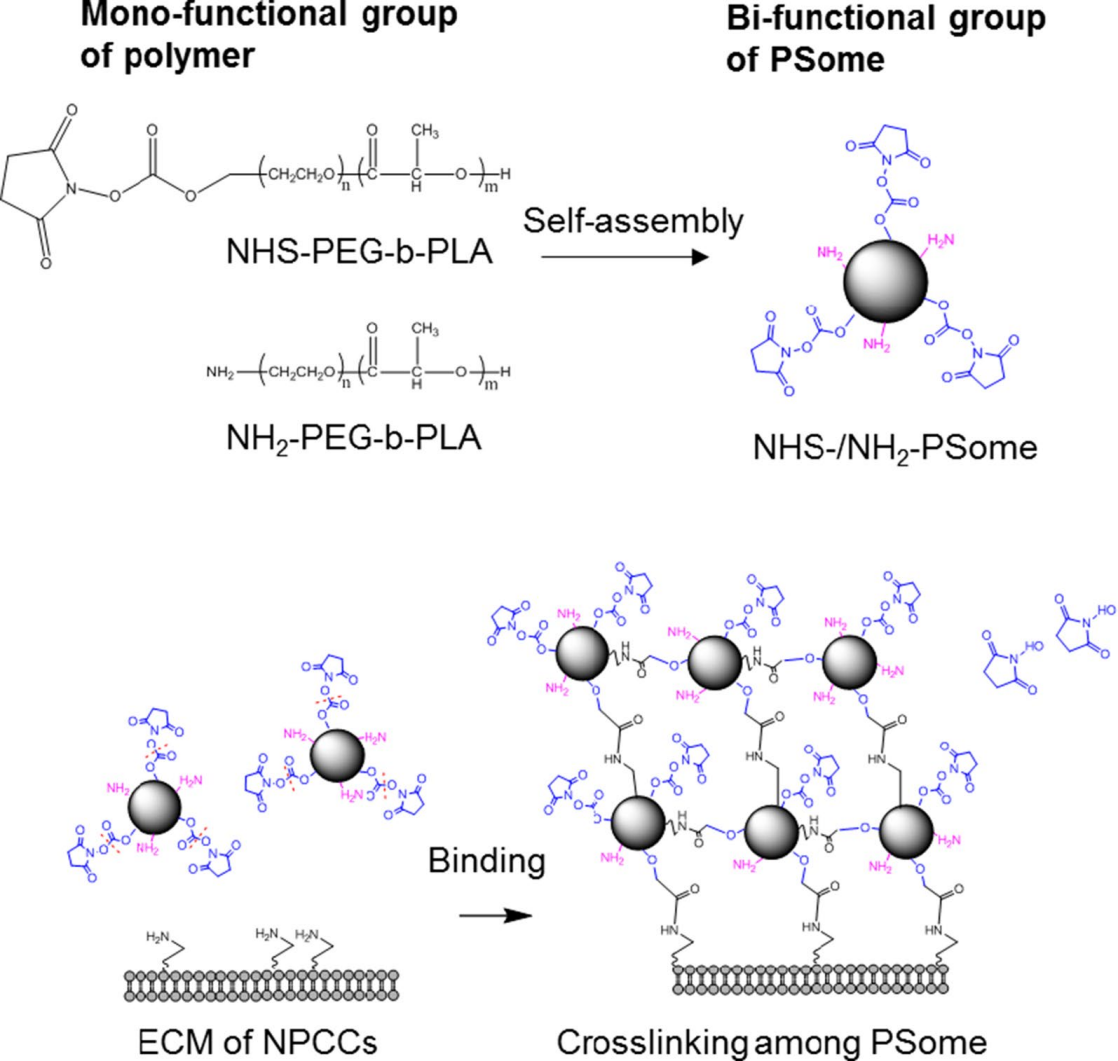
The illustration of improved stability of nano-encapsulation by cross-linking of PSomes

**Fig. 5:**
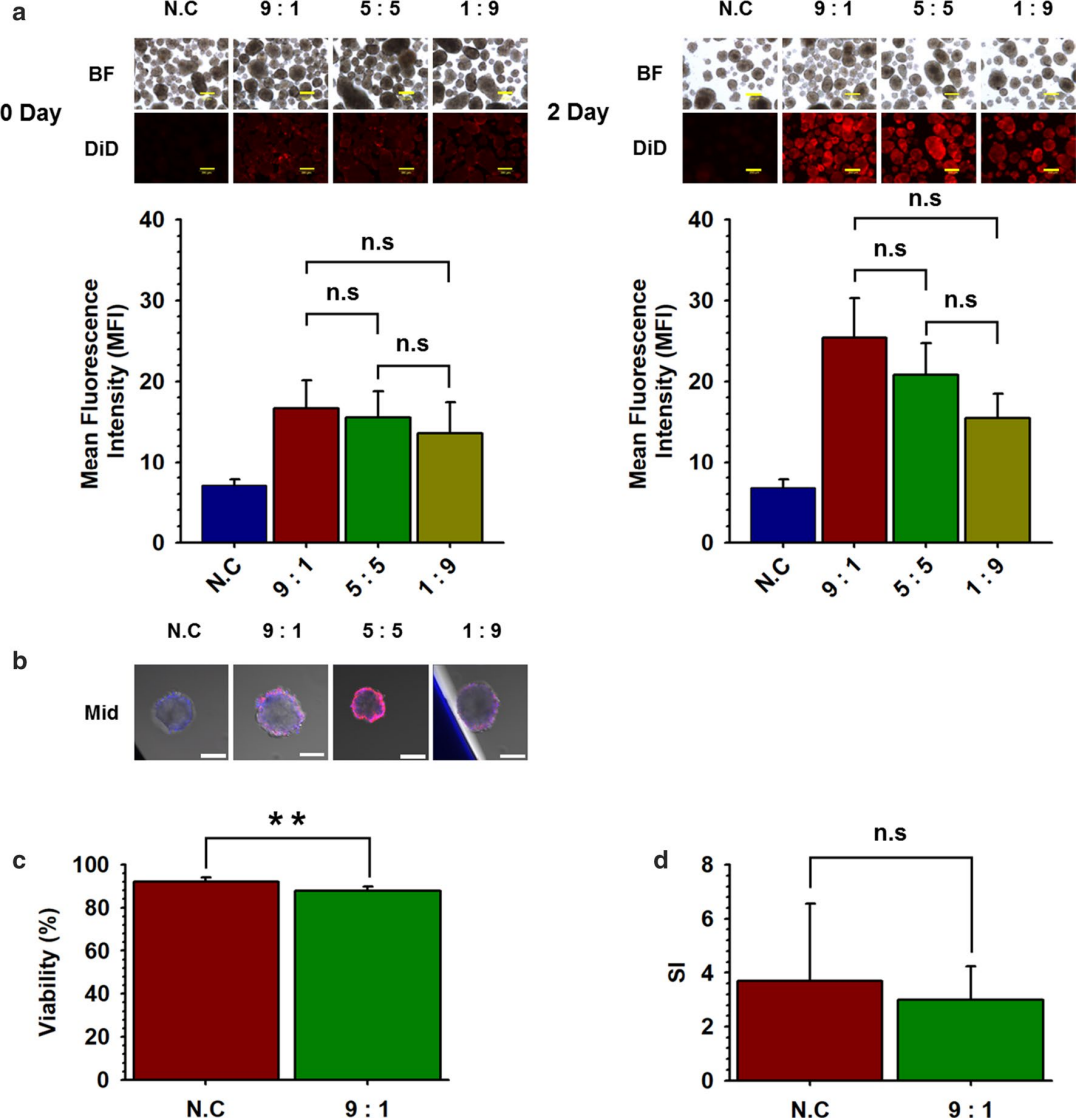
Coating efficiency and functionality of NHS-/NH_2_-PSome nano-encapsulated NPCCs. Coating efficiency and functionality of NHS-/NH_2_-PSome nano-encapsulated NPCCs. **a** DiD conjugated 9:1, 5:5, 1:9 of NHS-/NH_2_-PSome nano-encapsulated NPCCs (9:1, 5:5, 1:9) (NC; non-coated NPCCs). MFI shows the intensity of DiD-conjugated PSome nano-encapsulated NPCCs (*n* = 3) and NC control (*n* = 3). The scale bars represent 200 um; **b** CLSM of DiD-conjugated NHS-/NH_2_-PSome nano-encapsulated NPCCs (Mid; middle image using CLSM of DiD-conjugated NHS-/NH_2_-PSome nano-encapsulated NPCCs). Blue in Mid represent the cell through DAPI staining. The scale bars represent 100 um; C. Viability of 9:1 (*n* = 9) and NC control (*n* = 3). Data represent the mean ± S.D. **p < 0.01 versus NC; D. SI of 9:1 (*n* = 9) and NC control (*n* = 3). Data represent the mean ± S.D

To induce a more stable nano-encapsulation of NPCCs, we attempted to conjugate PSomes having two different functional groups. First, we conducted nano-encapsulation of the NPCCs by simultaneously adding different proportions of PSomes containing NHS and NH_2_ bifunctional groups (NHS-/ NH_2_-PSome) in one PSome (Scheme 1). Efficiency of nano-encapsulation was confirmed by the MFI of DiD conjugated in PSome nano-encapsulated NPCCs. Resulting images from fluorescence microscopy showed no significant differences among 9:1, 5:5, and 1:9 groups of NHS-/NH2-PSome nano-encapsulated NPCCs (9:1, 5:5, 1:9) on day 0 and day (Fig. 5a). However, in the CLSM results, the 5:5 group seemed to be over-coated and 1:9 showed insufficient coating while the 9:1 group formed a conformal coating, on day 1 (Fig. 5b). Therefore, we determined the optimal ratio of NHS-/NH2 in PSome to be 9:1. When functional assays were carried out for the 9:1 group, our results showed that the viability of the 9:1 group was significantly decreased when compared with that of the NC control (92.1%), but viability was maintained at normal levels (87.8%). The SI function of the 9:1 group was normal (3.0) when compared with that of the NC control (3.7) (Fig. 5c,d).

## Discussion

Diabetes mellitus, commonly known as diabetes, is a metabolic disease characterized by high blood glucose levels. Type 1 diabetes results from the failure of the beta cells in pancreas to produce enough insulin [16]. Transplantation of pancreatic islets containing insulin producing β cells has recently been used to cure type 1 diabetes. However, allo-islet transplantation is limited owing to shortage of donors; instead, xenotransplantation using islets from non-human animals has emerged as an alternative source of donor tissue. Owing to their physiological similarities to humans, ease of mass breeding, and availability of breeding in pathogen-free facilities, pigs are considered an optimal animal model for xeno-islet transplantation [1]. Especially, the NPCCs have been used valuably as good as the APIs. Although the maturity of NPCCs is lower than that of APIs, NPCCs have some advantages over APIs including, having a relatively simple and inexpensive islet isolation procedure, ability to develop resistance to hypoxic environments and proliferation in vivo after transplantation [1–3]. For these reasons, we used 3–5-day-old neonatal pigs as the islet source in this study.

Unfortunately, once pig islets are implanted into human or nonhuman primate blood vessels, severe immune reactions such as IBMIR or hyperacute rejection often occur. IBMIR usually occurs due to several tissue factors (TF) expressed in pig islets that mediate coagulation in human blood vessels via activation of an extrinsic pathway. Alpha-galactose or non-gal antigens expressed on the surface of pig cells can also be targets of natural human antibodies, followed by a complementing cascade activation called hyperacute rejection. As a result, grafts are lost following hypoxia by clot formation from the coagulation pathway and cell death by complement activation in the host [3]. To solve these problems, encapsulation, a method of coating pancreatic islets with biocompatible materials to protect them against attack by antibody or complement reactions, has been tried. First, macro-encapsulation uses a device with a semipermeable membrane containing the islet and is implanted next to blood vessels where it releases insulin into the blood stream in response to blood glucose levels [4]. Second, micro-encapsulation, mainly using alginate, has selective permeability and can allow oxygen and nutrients to pass through its porous surface, while blocking multiple cytokines and immune cell infiltration. However, because they use the same size of capsules regardless of the size of islets, it is difficult to conformally coat the islets. Additionally, fibrosis may occur, enclosing the graft after transplantation [17]. Lastly, surface modification of islets (nano-encapsulation) mainly uses polyethylene glycol (PEG) that have “stealth effect” property that blocks the interaction of materials coated with “stealth” polymer (PEG) and components in the blood (immune cells) in vivo [11, 18]. Our NPCCs nano-encapsulation strategy uses the modified PEG copolymers (PEG-b-PLA, Polymersome, PSome). Also, Psome has both hydrophilic and hydrophobic properties and can incorporate immunosuppressant or factors involved in cell differentiation or growth [8].

Nano-encapsulation of islets using PEG has been performed in basic HBSS buffer (pH 8.0 or above) to enhance the binding affinity between NHS on PEG and NH_2_ on ECM of islet [10–12]. However, since these conditions cannot provide suitable cell culture environment, we attempted nano-encapsulation in an environment mimicking the NPCC culture condition. To address the issues above, we tested plain F-10 media, NPCCs culture base medium, with physiological pH (without any supplements) used as the nano-encapsulation reaction buffer. Nano-encapsulation in F-10 with physiological pH showed a similar coating efficiency and maintained normal morphology of NPCCs when compared with the culture condition using basic HBSS buffer (Fig. 3). Therefore, we can propose a platform that minimizes NPCC damage during the nano-encapsulation in an NPCC culture mimicking environment.

Although the nano-encapsulation method for minimizing NPCC damage was established, the residual amount of NPCCs collected after nano-encapsulation decreased when the nano-encapsulation was performed in petri dishes. This means that you require more NPCCs for nano-encapsulation for transplantation. According to previous reports, islet yields were improved in some mouse strains by using BSA during isolation [19]. Also, BSA was used as a suspension culture by pre-coating the surface of cell culture dishes in rat hepatoma cell cultures [13]. Therefore, to increase the amount of NPCCs after nano-encapsulation, 0.25% BSA was added in F-10 nano-encapsulation reaction buffer, same to the concentration of BSA used for culturing NPCCs. As a result, the NPCC recovery rate increased significantly after nano-encapsulation (Fig. 4). This means that the correct number of islets (containing NPCCs) after the nano-encapsulation can be predicted and transplanted by minimizing islets (containing NPCCs) loss. In summary, this study using F-10 with 0.25% BSA for nano-encapsulation showed that (i) the normal morphology of NPCCs was maintained, (ii) the binding between NHS-conjugated PSome and NH2 in ECM of NPCCs was not interfered and (iii) the recovery rate of NPCCs after nano-encapsulation increased.

Finally, we attempted to enhance the stability of nano-encapsulation through (i) conjugation between PSomes and (ii) binding between the PSomes and ECM of NPCCs. First, NHS- and NH2-PEG-b-PLA polymers were mixed proportionally to form the bifunctional PSome (NHS-/NH2-PSome) that can bind to both PSome and ECM of NPCCs. We postulated that conjugation could be efficiently achieved by bifunctional groups within one PSome rather than conjugating two PSomes with mono-functional groups due to the potential interruption caused by binding between PSomes with the same functional group (NHS-NHS and NH2-NH2). As seen from the results, the conformal coating of NPCCs was achieved at a proportion of 9:1 of NHS-/NH2-PSome nano-encapsulated NPCCs, and viability and functionality were maintained (Fig. 5). However, further studies are needed to quantify the strength of the PSome bond, to prove that nano-encapsulation with bifunctional PSomes resulted in more stable encapsulation than that with the mono-functional PSome. Therefore, we suggest that our effective nano-encapsulation conditions mimicking the NPCC culture environment can be used in the nano-encapsulation strategy using islets containing NPCCs (Additional file 1).

## Conclusion

This study was conducted to determine an optimal method of nano-encapsulation of pancreatic islets (NPCCs) using PEG-based polymersomes (PSomes). First, using F-10 culture medium with pH of 7.3 can maintain the normal morphology of NPCCs after nano-encapsulation as compared with using basic HBSS buffer (pH 8.0), thereby minimizing damage to NPCCs during encapsulation. Second, adding 0.25% BSA to F-10 medium improved the yield of NPCCs by approximately 1.7 times following nano-encapsulation. Finally, we induced a more stable nano-encapsulation through the conjugation of bifunctional PSomes (NHS-/NH2-PSomes). The methods of nano-encapsulation presented in this paper may be applicable in nano-encapsulation of pancreatic islets using PEG-based nanoparticles.

## Supplementary Information

**Additional file 1.** **Supplement 1**. The morphologies and functionalities of NPCCs. A. The morphologies of NPCCs at day.1, 3, 5 after isolation (BF ; bright field). The scale bars represent 200 um; B. AO/PI staining of NPCCs at day.5 after isolation. AO stained the live cells (green) and PI stained the dead cells (red) (n=5). The scale bars represent 200 um; C: The viability of NPCCs was quantified from the result of AOPI staining at day.5 after isolation (n = 5); D: Stimulation index (SI) was calculated by dividing the insulin quantities at high glucose (28.0 mM) by that at low glucose (2.8 mM) at day.5 after isolation (n = 5). **Supplement 2**. The viability of THP-1 cell lines for polymersome. The viability of NHS-PSome nano-encapsulated THP-1 was measured through MTT assay (n = 4).

## Data Availability

Not applicable.

## Abbreviations

DiD: 1,1′-Dioctadecyl-3,3,3′,3′-tetramethylindodicarbocyanine, 4-chlorobenzenesulfonate salt
AO: Acridine orange
APIs: Adult porcine islets
BSA: Bovine serum albumin
BF: Bright field
CLSM: Confocal laser scanning microscopy
DAPI: 4′,6-Diamidino-2- phenylindole
DMSO: Dimethyl sulfoxide
DH2O: Distilled water
ELISA: Enzyme-linked immunosorbent assay
FBS: Fetal bovine serum
FITC: Fluorescein isothiocyanate
GSIS: Glucose-stimulated insulin secretion
HBSS: Hank’s balanced salt solution
IBMIR: Instant blood-mediated inflammatory reaction
IEQ: Islet equivalent
IBMX: Isobutylmethylxanthine
KRBB: Krebs–Ringer bicarbonate buffer
MFI: Mean fluorescence intensity
HEPES: N-(2-Hydroxyethyl)piperazine-N′-(2-ethanesulfonic acid)
NC: Negative control
NPCCs: Neonatal porcine islet like cell clusters
NHS: N-hydroxysuccinimide
NHP: Non-human primate
PLA: Poly lactide
PEG: Polyethylene glycol
PSomes: Polymersomes
PI: Propidium iodide
SI: Stimulation index
TF: Tissue factor
