# Supplementary material for: Optimization of Nano-encapsulation on Neonatal Porcine Islet-like Cell Clusters Using Polymersomes
Source: Nanoscale Res Lett. 2021 Mar 31;16:53. doi: 10.1186/s11671-021-03512-3 (PMC8012476; doi:10.1186/s11671-021-03512-3)
Supplement: Supplementary file 1 — Additional file 1. Supplement 1. The morphologies and functionalities of NPCCs. A. The morphologies of NPCCs at day.1, 3, 5 after isolation (BF ; bright field). The scale bars represent 200 um; B. AO/PI staining of NPCCs at day.5 after isolation. AO stained the live cells (green) and PI stained the dead cells (red) (n=5). The scale bars represent 200 um; C: The viability of NPCCs was quantified from the result of AOPI staining at day.5 after isolation (n = 5); D: Stimulation index (SI) was calculated by dividing the insulin quantities at high glucose (28.0 mM) by that at low glucose (2.8 mM) at day.5 after isolation (n = 5). Supplement 2. The viability of THP-1 cell lines for polymersome. The viability of NHS-PSome nano-encapsulated THP-1 was measured through MTT assay (n = 4). [file 11671_2021_3512_MOESM1_ESM.docx]

**Optimization of nano-encapsulation on neonatal porcine islet-like cell clusters using polymersomes**

**Running title : Nano-encapsulation of islets**

**Authors**

**Sang Hoon Lee^1*^, Hyun-Ouk Kim^2, 3*^, Jung-Taek Kang^1†^**

^1^ MGENPLUS Biotechnology Research Institute, Mgenplus Co., Ltd., Seoul, 06688, Republic of Korea

^2^Department of Biotechnology and Bioengineering, Kangwon National University, Chuncheon, Gangwon-do, 24341, Republic of Korea

^3^Biohealth-machinery Convergence Engineering, Kangwon National University, Chuncheon, Gangwon-do, 24341, Republic of Korea

* These authors contributed equally.

**^†^ Corresponding author: Dr. Jung-Taek Kang**, MGENPLUS Biotechnology Research Institute, Mgenplus Co., Ltd., Seoul, 06688, Republic of Korea

**Email: exodus78@mgenplus.com**

**Tel: 82-70-5083-6422**

**Figure legends**

**Supplement 1 The morphologies and functionalities of NPCCs.** A. The morphologies of NPCCs at day.1, 3, 5 after isolation (BF ; bright field). The scale bars represent 200 um; B. AO/PI staining of NPCCs at day.5 after isolation. AO stained the live cells (green) and PI stained the dead cells (red) (n=5). The scale bars represent 200 um; C: The viability of NPCCs was quantified from the result of AOPI staining at day.5 after isolation (n=5); D: Stimulation index (SI) was calculated by dividing the insulin quantities at high glucose (28.0 mM) by that at low glucose (2.8 mM) at day.5 after isolation (n=5).

**Supplement 2 The viability of THP-1 cell lines for polymersome.** The viability of NHS-PSome nano-encapsulated THP-1 was measured through MTT assay (n=4).


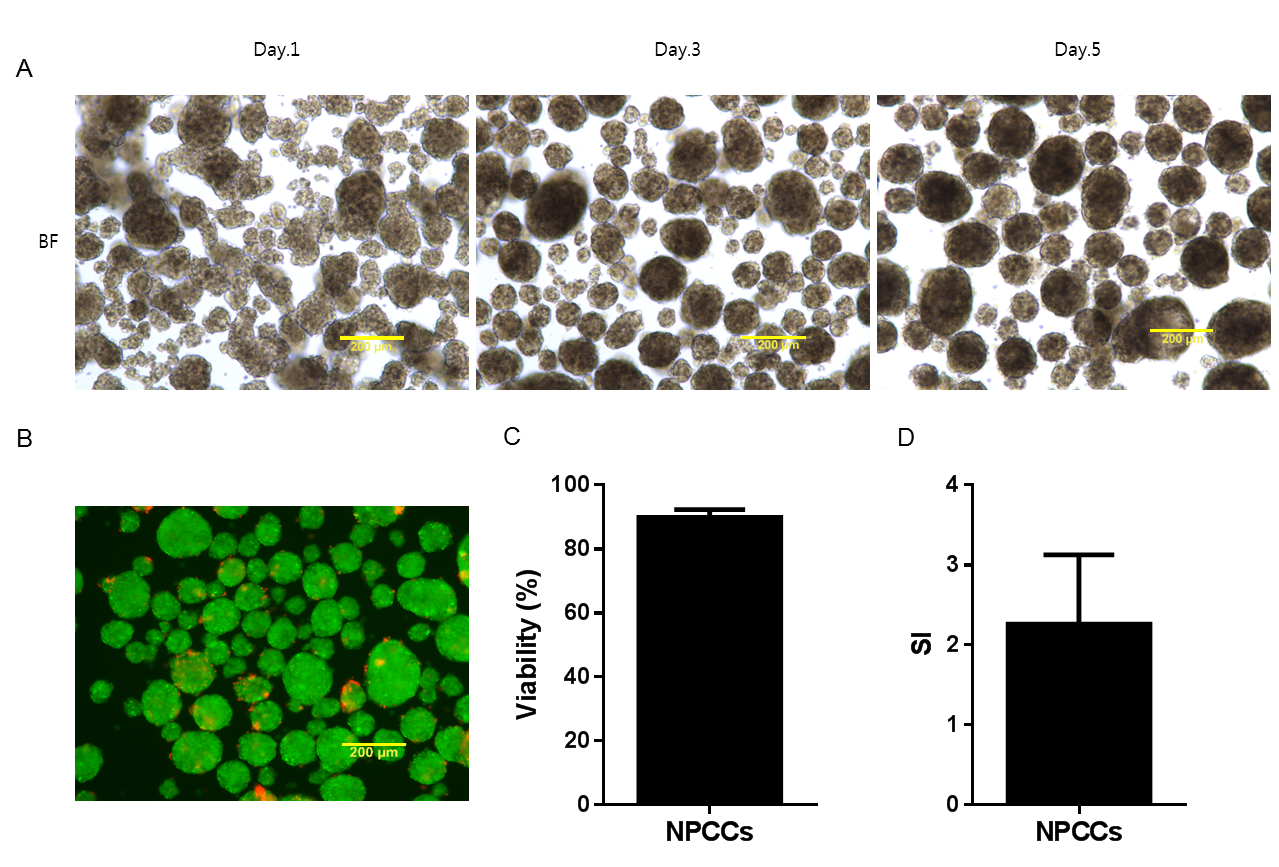


**Supplement 1 The morphologies and functionalities of NPCCs .** A. The morphologies of NPCCs at day.1, 3, 5 after isolation (BF ; bright field). The scale bars represent 200 um; B. AO/PI staining of NPCCs at day.5 after isolation. AO stained the live cells (green) and PI stained the dead cells (red) (n=5). The scale bars represent 200 um; C: The viability of NPCCs was quantified from the result of AOPI staining at day.5 after isolation (n=5); D: Stimulation index (SI) was calculated by dividing the insulin quantities at high glucose (28.0 mM) by that at low glucose (2.8 mM) at day.5 after isolation (n=5).

**Supplement 2 The viability of THP-1 cell lines for polymersome.** The viability of NHS-PSome nano-encapsulated THP-1 was measured through MTT assay (n=4).
